# Green Synthesis of Mesquite-Gum-Stabilized Gold Nanoparticles for Biomedical Applications: Physicochemical Properties and Biocompatibility Assessment

**DOI:** 10.3390/polym15173533

**Published:** 2023-08-24

**Authors:** Ana M. Pinilla-Torres, Celia N. Sanchez-Dominguez, Karla Basilio-Bernabe, Paola Y. Carrion-Garcia, Jorge A. Roacho-Perez, Elsa N. Garza-Treviño, Hugo Gallardo-Blanco, Margarita Sanchez-Dominguez

**Affiliations:** 1 Grupo de Química Coloidal e Interfacial Aplicada a Nanomateriales y Formulaciones, Centro de Investigación en Materiales Avanzados, S.C. (CIMAV, S.C.), Unidad Monterrey, Apodaca 66628, Mexico; ana.pinilla@cimav.edu.mx (A.M.P.-T.);; 2 Departamento de Bioquímica y Medicina Molecular, Facultad de Medicina, Universidad Autónoma de Nuevo León, Monterrey 64460, Mexico; celia.sanchezdm@uanl.edu.mx (C.N.S.-D.); carriongarcia.paola@gmail.com (P.Y.C.-G.); alberto.roachoprz@uanl.edu.mx (J.A.R.-P.); elsa.garzatr@uanl.edu.mx (E.N.G.-T.); 3 Departamento de Genética, Facultad de Medicina, Universidad Autónoma de Nuevo León, Monterrey 64460, Mexico

**Keywords:** mesquite gum, gold nanoparticles, green synthesis

## Abstract

Using cytotoxic reducing and stabilizing agents in the synthesis of gold nanoparticles (AuNPs) limits their use in biomedical applications. One strategy to overcome this problem is using “green” synthesis methodologies using polysaccharides. In the present study, we propose a green methodology for synthetizing AuNPs with mesquite gum (MG) as a reducing agent and steric stabilizer in Gold(III) chloride trihydrate aqueous solutions to obtain biocompatible nanoparticles that can be used for biomedical applications. Through this method, AuNPs can be produced without using elevated temperatures or pressures. For synthetizing gold nanoparticles coated with mesquite gum (AuNPs@MG), Gold(III) chloride trihydrate was used as a precursor, and mesquite gum was used as a stabilizing and reducing agent. The AuNPs obtained were characterized using UV-Vis spectroscopy, dynamic light scattering, transmission electron microscopy, scanning transmission electron microscopy, and FT-IR spectroscopy. The stability in biological media (phosphate buffer solution), cytotoxicity (MTT assay, hematoxylin, and eosin staining), and hemocompatibility (Hemolysis assay) were measured at different concentrations and exposure times. The results showed the successful synthesis of AuNPs@MG with sizes ranging from 3 to 30 nm and a zeta potential of −31 mV. The AuNPs@MG showed good colloidal stability in PBS (pH 7.4) for up to 24 h. Finally, cytotoxicity assays showed no changes in cell metabolism or cell morphology. These results suggest that these gold nanoparticles have potential biomedical applications because of their low cytotoxicity and hemotoxicity and improved stability at a physiological pH.

## 1. Introduction

Gold nanoparticles (AuNPs) are widely used in biomedical applications such as vehicles for gene therapy, contrast agents, photothermal therapy, and drug delivery [1,2,3,4,5] due to their unique properties and their size- and shape-related optical properties, large surface-to-volume ratio, and biocompatibility [6,7]. These AuNPs have been synthesized through different methodologies, among which the most common is the chemical reduction method. In this approach, metal ions from their ionic salts are reduced using various chemical reducing agents in the presence of a stabilizing agent under specific reaction parameters, such as pH and temperature [8]. In some cases, the reducing agent also acts as a AuNP stabilizer [9,10]. Sodium citrate is one of the most widely used reducing and stabilizing agents (Turkevich method) [11]. However, in recent years, there has been a growing preference for using green reducing agents in the synthesis of AuNPs due to their numerous advantages, such as excellent stabilization ability, lack of toxicity, and low cost [12,13]. Green reducing agents offer distinct benefits, making them appealing alternatives to traditional chemical agents. Their excellent stabilization ability ensures uniform and stable nanoparticle formation, while their eco-friendly nature reduces environmental impact and promotes sustainable nanomaterial synthesis. Examples of green reducing agents include plant extracts, phytochemicals, microorganisms, and polysaccharides, such as Arabic Gum [14,15,16,17,18,19,20]. This gum is the complex exudate of the *Acacia senegal* and *Acacia seyal* trees, and it has great relevance in the pharmaceutical and food industries [21].

Additionally, this gum has been used to synthetize gold nanoparticles for biomedical applications [18,19]. In the process of AuNP synthesis, Arabic gum (AG) has been used as a stabilizing agent in the presence of different reducing agents (sodium citrate, sodium borohydride, peptides, and ionic liquids) [18,19,22,23]. Its functional groups (mainly carboxylate and amine) easily interact with the surface of AuNPs, conferring steric and electrostatic stability to the AuNPs [23]. Additionally, AG can prevent AuNP aggregation under biological conditions, improving AuNP biocompatibility [22].

Previously, our research group successfully employed mesquite gum (MG) as a stabilizing and reducing agent for silver nanoparticles [24]. MG is derived from *Prosopis velutina* (a mesquite tree species), is a proteinaceous arabinogalactan gum, and shares a similar structure and properties with Arabic Gum (AG), a well-known nanoparticle stabilizing agent. The *Prosopis* genus comprises approximately 44 species, including 10 mesquite species, primarily distributed across Mexico and the southern United States [25]. MG comprises L-arabinose, D-galactose, 4-O-Methyl-D-glucuronic acid, and L-rhamnose; its molar mass is 484,000 g/mol [26]. Its functional properties are closely tied to its structure, influencing solubility, viscosity, and emulsification capacity.

One of the distinctive features of MG is its relatively low viscosity; even at high concentrations compared to AG, the MG proteins can decrease the surface tension to operate as a steric stabilizer [27]. Moreover, MG contains proteins (3–7%) mainly composed of hydroxyproline, glycine, valine, and serine [26,27]. These MG proteins play a vital role in reducing the surface tension and acting as steric stabilizers, making MG an excellent candidate for “green” synthesis methods of silver nanoparticles [24]. 

The beneficial effects of incorporating the MG branched polyethyleneimine (b-PEI) structure have been demonstrated in a previous study. This integration decreases b-PEI hemotoxicity and improves its buffer capacity, making it a promising non-viral vector for gene therapy applications [28]. Beyond its potential in gene therapy, MG’s unique properties and eco-friendly nature hold promise in various fields. For instance, MG-based nanoparticle synthesis can find applications in drug delivery systems, nanomedicine, catalysis, and environmental remediation. Its biocompatibility and sustainable origin further add to its appeal as a versatile material in nanotechnology.

The present study aims to synthesize AuNPs using mesquite gum (MG) as a reducing and stabilizing agent and subsequently evaluate their cytotoxicity and hemocompatibility. The motivation behind this research is to develop a mild and eco-friendly synthesis method for AuNPs without the need for additional stabilizing or reducing agents.

The characterization of the AuNPs@MG synthetized was achieved using various techniques, including UV-Vis spectroscopy, scanning transmission electron microscopy (STEM), dynamic light scattering (DLS), zeta potential analysis, transmission electron microscopy (TEM), and Fourier Transform Infrared (FTIR) spectroscopy. These characterization methods allowed us to gain valuable insights into the size, morphology, surface charge, and structural properties of the synthesized AuNPs.

Furthermore, the stability of the nanoparticles in biological media, specifically phosphate-buffered saline (PBS), was evaluated to assess their potential for biomedical applications. Understanding the behavior of AuNPs@MG in a relevant biological environment is crucial for their successful translation into practical applications.

This study also includes an assessment of the cytotoxicity of AuNPs@MG using the MTT assay, providing essential information about their biocompatibility and potential impact on cell viability. Additionally, hematoxylin and eosin (H&E) stains and a hemolysis assay were performed to evaluate the nanoparticles’ effect on blood cells, providing insights into their hemocompatibility.

## 2. Materials and Methods

### 2.1. Materials

Local suppliers manually collected mesquite gum samples from *Prosopis velutina* trees as exudate pearls in the Mexican state of Sonora [28]. Basically, mesquite gum samples were collected manually from the *Prosopis velutina* tree in the state of exudate pearls by suppliers located in Sonora, Mexico [28]. Thus, a batch of mesquite gum pearls was acquired from a local convenience store, specifically “Mieles de Sonora” in Hermosillo, Mexico. To ensure its suitability for AuNP synthesis, the purchased mesquite gum underwent purification at the laboratory [28].

### 2.2. Mesquite Gum Purification

The mesquite gum purification methodology described by Moreno-Trejo et al. [24] was employed to obtain purified mesquite gum to synthetize AuNPs. First, the mesquite exudate pearls were carefully chosen and cleaned using established methods; basically, the lighter pearls were selected, while the darker pearls, rich in tannins, were discarded from the further process, and then any little sticks from the mesquite tree and any present dirt were mechanically removed from the lighter pearls [29,30]. Subsequently, the cleaned pearls were pulverized using a mortar. The resulting powder was then dissolved in distilled water at room temperature and left to hydrate for 24 h. After the hydration period, the liquid was filtered using a Whatman no. 2 filter paper to remove impurities. The filtered solution was then frozen for 15 h and subsequently lyophilized using a FreeZone freeze dryer (Labconco, Kansas City, MO, USA) for 26 h [28].

### 2.3. Synthesis of Gold Nanoparticles Using Mesquite Gum (AuNPs@MG)

Different weight ratio *W_MG_*/*W_Au_* (1:15, 1:26, and 1:50) proportions were used in the experiment. The specific volume of MG was added to 5 mL of the aqueous solution of 1 mM HAuCl_4_ · 3H_2_O at room temperature (99.9% Sigma-Aldrich, St. Louis, MO, USA) with gentle stirring. The reaction mixtures were first vortexed and then kept under continuous stirring for 180 min at 70 °C using a water bath. Finally, AuNPs@MG were centrifuged for 20 min at 10,000 rpm and washed with 5 mL of deionized water (twice). After this process, AuNPs@MG were redispersed in deionized water for further use and characterization.

### 2.4. Characterization Techniques

The UV–visible absorption spectra of the colloidal dispersions containing AuNPs@MG were recorded using a UV–visible spectrometer (Optizen POP, Mecasys, Daejeon, Republic of Korea) within the 300–800 nm range. The zeta potential of the AuNPs@MG colloidal dispersions (in water) was determined experimentally with electrophoretic mobility; the calculation of zeta potential value was conducted with software applying the Henry’s equation (Smoluchowski approximation), using a Zetasizer Nano ZS (Malvern Instruments, Malvern, UK). The hydrodynamic diameter of the AuNPs@MG colloidal dispersions in water was calculated with dynamic light scattering (DLS), utilizing a Zetasizer Nano ZS (Malvern Instruments, Malvern, UK); the automatic algorithm, which uses a combination of monomodal (cumulants), non-negative least squares (NNLS), and CONTIN algorithms, was used for data analysis. The morphology and size were analyzed with transmission electron microscopy (TEM) using a JEOL JEM 2200 FS (JEOL Ltd., Tokyo, Japan), as well as with scanning transmission electron microscopy (STEM) using a Nova Nano 200 FEI (FEI Company, Eindhoven, The Netherlands). FTIR analysis was performed utilizing a Thermo Nicolet 6700 FT-IR (Thermo Fisher Scientific, Waltham, MA, USA) [28].

### 2.5. Preliminary Assessment of the Stability of AuNPs in Biological Media

The colloidal stability of AuNPs in biological media is crucial for their use in biomedical applications. For this assay, AuNPs@MG were separated by centrifugation at 10,000 rpm for 20 min, and after that, they were dispersed in 1 mL of PBS (pH 7.4). Colloidal stability was monitored after keeping the dispersions at room temperature for 24 and 48 h.

### 2.6. Biological Assays

#### 2.6.1. Cytotoxicity: MTT Assay

The MTT assay evaluated the biocompatibility of AuNPs@MG by cell viability in the fibroblast 3T3L1 cell line from mouse embryo (ATCC, Manassas, VA, USA). A total of 5000 3T3L1 cells were deposited per well in a 96-well microplate and subsequently cultured with DMEM, with 10% fetal bovine serum supplemented (both components acquired from Thermo Fisher Scientific, Waltham, MA, USA), and 1% of Penicillin–Streptomycin (Thermo Fisher Scientific, Waltham, MA, USA) [28].

Cells were treated with different concentrations of the AuNPs@MG in triplicate (4.5, 2.25, 1.11, 0.56, and 0.28 µg/mL. At 24, 48, and 72 h of exposition time, cells were incubated with 100 µg/mL MTT diluted in supplemented DMEM for 4 h. After incubation, the medium was carefully retired, and then 50 µL of isopropanol (acidified at pH 3 with a few drops of dilute HCl) was carefully added to dissolve the formazan. Absorbance was registered at 570 nm. Cell viability was estimated using the 100% control viability sample of non-exposed cells.

#### 2.6.2. Hematoxylin and Eosin (H&E) Stains

Hematoxylin and eosin (H&E) stain was used to evaluate the morphology of the fibroblast 3T3L1 cells, which had been exposed to different concentrations of AuNPs@MG (4.5, 1.11, and 0.28 µg/mL); for comparison, cells were also exposed to the same concentrations of MG. A previously reported protocol was followed [31]. First, the culture medium was carefully extracted, and cells were PBS-buffer-washed (Thermo Fisher Scientific, Waltham, MA, USA) three times. Subsequently, for the cell fixation, we added 50 µL of cold methanol to each plate well and incubated at −20 °C for 10 min. The 3T3L1 cells were washed three times with PBS buffer. For the staining step, the 3T3L1 cells were incubated at room temperature for 5 min in hematoxylin solution, followed by an HCl (diluted at 0.5% in ethanol) distilled water wash, 5 min of incubation in eosin, and a tap water wash. The 3T3L1 cell morphology was analyzed with an inverted microscope CKX41 (Olympus, Shinjuku, Japan) [28]. 

#### 2.6.3. Statistical Analysis

For the statistical analysis of MTT absorbance results, the 3T3L1 cells without treatment were used as 100% of viability reference. The cell viability percentages of treatment assays were interpreted with an analysis of variance (ANOVA) and then with a Tukey’s HSD test (Honestly Significant Difference); we used a 95% confidence interval to determine significant differences by comparison to the control group [28].

## 3. Results and Discussion

**Figure 1:**
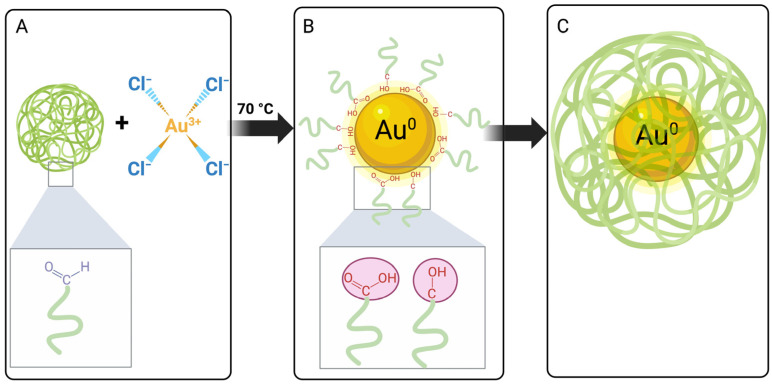
Schematic diagram of the proposed Au reduction reaction: (**A**) initial reactants; (**B**) interaction of carboxylic acid and alcohol groups from MG onto AuNP’s surface for stabilization; (**C**) overall scheme of AuNPs@MG sterically stabilized. Created with BioRender.com.

Many authors have documented the synthesis of AuNPs utilizing AG as a stabilizing agent and different reducing agents [17,18,19,22,23] (Table S1). AG has satisfied the demand from the food and pharmaceutical industry sectors for a long time; however, over the last few years, droughts in the regions where it grows have caused supply problems. The shortage in gum generates an increase in its cost, whereby it is necessary to find a substitute with similar properties. A possible substitute could be mesquite gum since its structure and properties are similar to AG’s. This work demonstrated that MG could be utilized as a reducing and stabilizing agent in the preparation of AuNPs. A schematic diagram of the proposed reduction reaction is shown in Figure 1.

The gum solution is heated, which allows the biopolymer to expand, adopting a conformation in which the functional groups of the mesquite gum are more sterically accessible to interact with gold ions. Subsequently, when adding the gold solution, the presence of Au^3+^ ions in the reaction medium leads to the oxidation of -CHO groups (present in polysaccharides) to -COOH groups. In addition, since the process is carried out at 70 °C, alcohol groups, which are abundant in polysaccharides such as MG, could also participate in the reduction reaction. Such alcohol groups would be oxidized to carbonyls (ketones or aldehydes) or carboxylic acids, depending on their primary or secondary nature. Parallel to this oxidation process, the Au^3+^ ions are reduced to Au^0^ (Figure 1). Finally, the generated AuNPs are stabilized by the interaction with the oxygen atoms from the hydroxyl and carbonyl groups present in MG [32,33,34].

### 3.1. UV-Vis Spectroscopy

**Figure 2:**
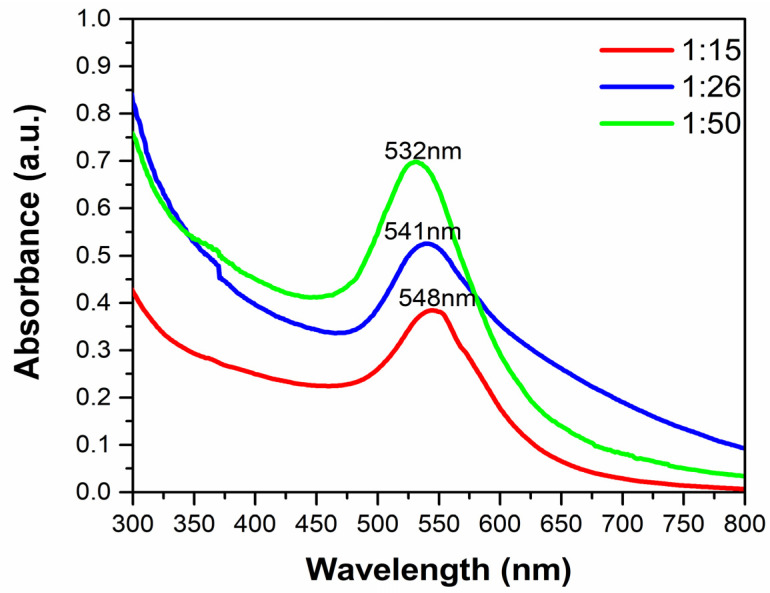
UV–Vis spectrum of the AuNPs@MG at three different weight ratios (*W_MG_*/*W_Au_*).

The AuNPs@MG were characterized through UV–Vis spectroscopy. Figure 2 illustrates the UV–Vis absorption spectra of Au colloid solutions obtained from the three different weight ratios (1:15, 1:26, and 1:50) with a gold precursor concentration of 1.0 mM.

Figure 2 shows a shift in the absorption maximum towards shorter wavelengths (548 to 541 nm) when passing from the 1:15 to the 1:26 ratio. A similar displacement was observed when comparing the 1:26 and 1:50 ratios (541 to 532 nm). These shifts in the absorption maximum when increasing the amount of MG have been reported by other authors who have used Arabic gum as a stabilizing and reducing agent [5,17]. The variation in optical properties might be due to the difference in their particle sizes and size distribution. As the size of gold particles decreases, the absorption maxima shift towards smaller wavelengths [35,36]. Thus, these results suggest that as the amount of MG used for synthesis is higher, at a constant precursor concentration, the particle size becomes smaller since a higher excess of reducing/stabilizing agent results in more favorable nucleation and decreased growth, as well as improved stabilization leading to a particle size distribution shifted towards a smaller size.

### 3.2. Dynamic Light Scattering (DLS)

**Figure 3:**
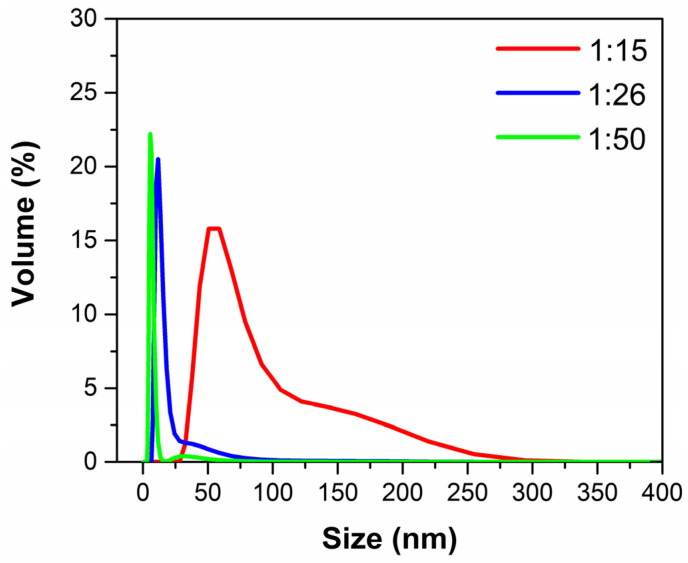
Size distribution of AuNPs@MG at three different weight ratios.

The AuNPs@MG hydrodynamic diameter was calculated using the dynamic light scattering (DLS) technique. Table 1 shows the hydrodynamic diameter (volume %) and PdI values obtained in the three conditions evaluated. In the case of the 1:15 and 1:26 ratios, we observed only one particle size population (monomodal), while for the 1:50 ratio, we observed two populations (bimodal). The monomodal size population may be related to the excess of MG in the 1:50 ratio; excess gum may form aggregates on its own, similar to Arabic gum, given their hydrocolloid nature [37]. However, by inspecting Figure 3 and Figure S1, which show the particle size distribution for the samples at three different weight ratios (*W_MG_*/*W_Au_*), it can be observed clearly that the second population of the AuNPs@MG in the 1:50 ratio sample is very small, and the main peak, centered at 5.43 nm, is very narrow. In contrast, the peaks of the samples with 1:15 and 1:26 ratios are wider and not Gaussian, suggesting the formation of agglomerates or clusters of nanoparticles. Thus, as suggested by the UV-Vis spectroscopy results, a decrease in the hydrodynamic diameter with the increase in the amount of MG used in the synthesis was observed. This may arise because by having a more significant amount of MG, the Au^3+^ ion reduction process and the stabilization of the generated particles is faster and more efficient, thus avoiding secondary growth processes that could form larger particles.

### 3.3. Zeta Potential

To determine the AuNPs@MG surface charge, zeta potential measurements were carried out (Table 2). In the three ratios evaluated for the synthesis, the zeta potential values were negative. The origin of the AuNPs@MG negative charges is due to the MG-ionizable groups, such as carboxyl, from the acidic monosaccharides and amino acids from the protein fractions. With a pH of 4.6 as the experimental condition, the carboxyl groups are dissociated [23], resulting in a negatively charged surface of AuNPs@MG. The obtained values are compatible with stable particles in an aqueous solution (values between + 30 and −30 mV). Similar zeta potential values were obtained by other authors [18,23] who synthesized AuNPs using AG, which presents a structure and properties similar to MG.

### 3.4. Transmission Electron Microscopy

Based on the results from the characterizations performed on the AuNPs@MG with three different HauCl_4_: GM weight ratios (UV-Vis spectroscopy, DLS, and zeta potential), one of the samples was selected, taking into account parameters such as the polydispersity index and a monomodal particle size distribution from hydrodynamic diameter results, as well as the highest value of zeta potential. The selected weight ratio was 1:15.

**Figure 4:**
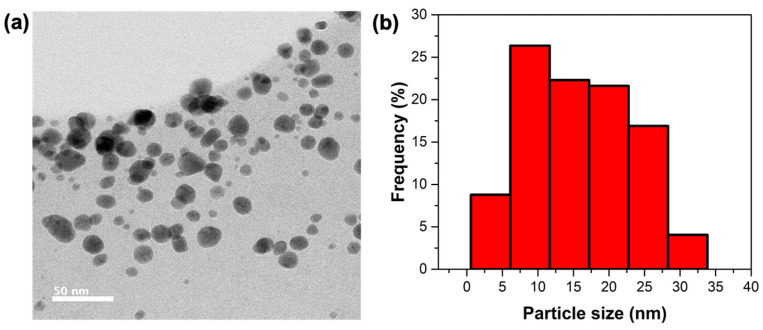
(**a**) TEM micrograph of AuNPs@MG obtained at a 1:15 weight ratio. (**b**) Particle size distribution histogram (TEM) using ImageJ Software, version 1.54d (scale bar 50 nm).

Figure 4 shows a TEM micrograph of the AuNPs obtained under this condition and their respective size histogram. The particles have a globular morphology with a mean diameter of 18.3 ± 7.3 nm (Figure 4 and Figure S1 shows additional STEM images). The size and morphology obtained are adequate since globular particles in this size range can cross biological barriers and do not cause damage to blood vessels [38]. In addition, these particles are not too small, such as those of the 1:50 ratio sample (around 5 nm hydrodynamic radius), which may cause rapid clearance from the body [39].

### 3.5. FTIR Spectroscopy

**Figure 5:**
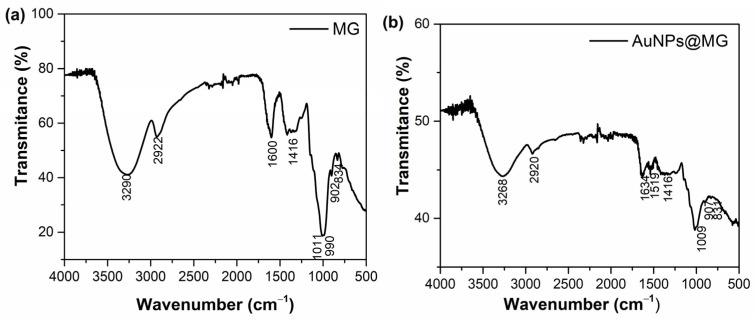
FTIR spectra of (**a**) Mesquite gum and (**b**) AuNPs@MG (1:15).

The functional groups of MG involved in reducing gold ions were evidenced using FTIR spectroscopy (Figure 5 and Table 3). With MG’s FTIR spectra, we identified O-H and C-H functional group bands at 3290 cm^−1^ and 2922 cm^−1^ [28]. The 1600 cm^−1^ band is set to amide I, attributed to the MG glycoprotein component; at 1416 cm^−1^, the COO- asymmetric stretching bands were recognized. The 1011 cm^−1^ and 990 cm^−1^ bands can be assigned to C-O and the C-O-H functional groups of carbohydrates (mannose, glucose, and galactose) [28]. The 834 cm^−1^ band corresponds to pyranose glycosidic acetal groups. Moreno-Trejo et al. reported the first characterization results of purified MG utilized to synthesize silver nanoparticles and stabilize essential citrus oil nanoemulsions [24,28,40].

For the AuNPs@MG spectrum, a shift in most of the peaks and, in some cases, a decreased intensity was observed (Figure 5) from 3290 to 3268 cm^−1^ (O-H stretching), 1600 to 1634 cm^−1^ (C-O stretching and N-H bending), and 1011 to 1009 cm^−1^, indicating the gold binding with hydroxyl and carboxylate groups. Based on the displacement of the bands corresponding to the hydroxyl and carbonyl groups, it is proposed that the hydroxyl and carbonyl groups of the MG are probably implicated in the reduction reaction to produce AuNPs@MG. These variations in the wavenumber values of the carboxylate and hydroxyl groups have been previously reported in other works in which gold nanoparticles were synthesized using AG [17,41] and our previous report on the synthesis of silver nanoparticles using MG [24].

It has also been reported by Kuhn that a band around 1613 cm^−1^ was shown by periodate-oxidized methyl α-D-glucopyranoside, and it was assigned to aldehydic carbonyl; furthermore, periodate-oxidized cellulose shows only a very weak band, arising from the hemialdal -CH-(OH)-O-CH(OH)- formed by the hydration of two aldehyde groups per oxidized residue [42]. The weakening and shift of the band from 1600 to 1634 cm^−1^ could thus also arise from aldehyde groups which were oxidized during the reaction with gold ions, which in turn were reduced to Au^0^.

**Table 3:** FTIR peaks assignment for MG and AuNPs@MG (1:15).

MG [24,28]		AuNPs@MG (1:15)	
Peak Position (cm^−1^)	Peak Assignment	Peak Position (cm^−1^)	Peak Assignment
3290	O-H	3268	O-H
2922	C-H	2920	C-H
------	------	1539	COO- asymmetric stretching [43].
1600	Amide I (stretching of the C=O and C-N).	1634	Amide I (stretching of the C=O and C-N) [44].
1416	COO- symmetric stretching.	1416	COO- symmetric stretching.
1011	C-O and the C-O-H groups of carbohydrates (such as glucose, mannose, and galactose).	1009	C-O and the C-O-H groups of carbohydrates (such as glucose, mannose, and galactose).
990	C-O and the C-O-H groups of carbohydrates (such as glucose, mannose, and galactose).		
902	C-O and the C-O-H groups of carbohydrates (such as glucose, mannose, and galactose).	907	C-O and the C-O-H groups of carbohydrates (such as glucose, mannose, and galactose).
834	Pyranose glycosidic acetal groups.	831	Pyranose glycosidic acetal groups.

### 3.6. Evaluation of the Stability of Gold Nanoparticles at Physiological pH

Evaluating the stability of AuNPs in biological media is important because when particles are introduced into the body or a biological environment, the NPs interact with complex biological environments with components such as platelets, proteins, antibodies, blood components, extracellular matrix, cytoplasm, cell organelles, and nucleic acids [45]. In addition, blood or physiological media contain serum proteins, electrolytes, and metabolites that contribute to a high ionic strength. Consequently, these complex biological interactions can affect some biomolecules and cellular components [46] and reduce the NPs’ stability in the biological environment.

For this purpose, PBS was used to simulate a biological medium, and water was used as a control. UV–Vis absorption spectra of AuNPs@MG (1:15) dispersed in PBS were taken at 0, 24, and 48 h.

**Figure 6:**
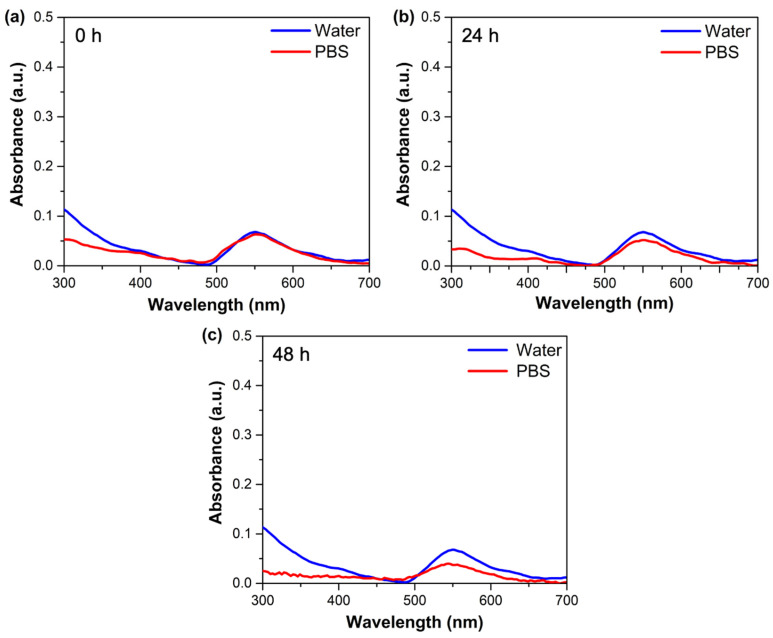
UV-Vis absorption spectra of AuNPs@MG (1:15) after (**a**) 0 h, (**b**) 24 h, and (**c**) 48 h incubation in water and phosphate-buffered saline (PBS).

In the case of AuNPs@MG dispersed in PBS, no significant changes were observed in the maximum absorption band, the shape of the band, or its intensity after 24 h of incubation (Figure 6a,b); however, after 48 h of incubation, some decrease in the band intensity was observed, which could be due to the partial agglomeration of the nanoparticles (Figure 6c). The stability of AuNPs@MG in this media is associated with mesquite gum on the AuNPs@MG surface, which protects them from aggregation. The natural gums carry specific functional groups, such as -OH, -NH_2_, -CHO, -CONH_2_, and -COOH, which can contribute to the surface stabilization of NPs better than other green synthesis approaches [47]. Previous studies have shown the ability of natural gums to stabilize AuNPs [41,48]. 

### 3.7. Biological Assays

#### 3.7.1. Hemolysis Assay

**Figure 7:**
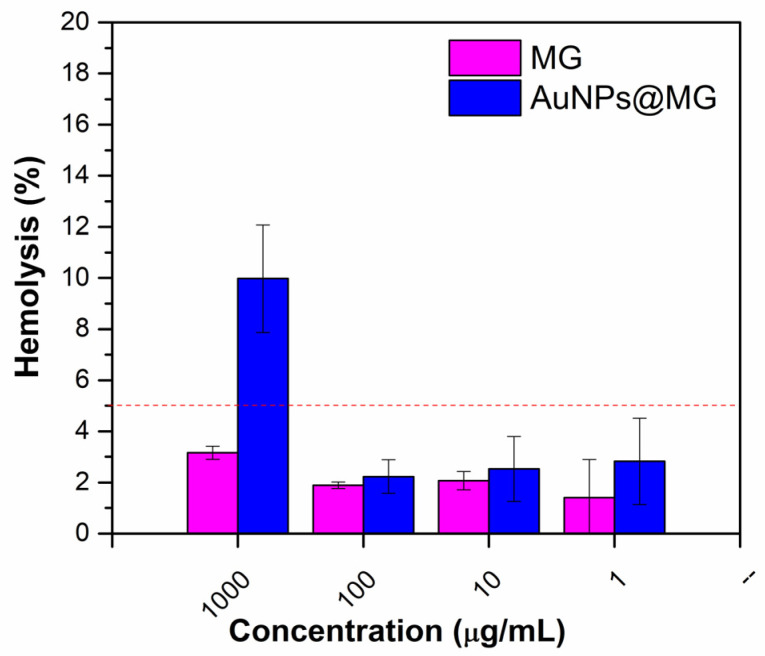
MG and AuNPs@MG (1:15) hemolysis assay results. Dotted red line indicates the upper limit of hemolysis corresponding to “slightly hemolytic” classification (2–5% hemolysis).

The hemolysis test can provide hemocompatibility evidence of the potential side effects of nanomaterials intended for intravenous administration [49]. The quantitative assay measures the hemoglobin released from the potentially lysed red blood cells exposed to the nanomaterials [31]. To our knowledge, this work is the first to report the hemocompatibility of AuNPs@MG. For this assay, concentrations of 1000, 100, 10, and 1 µg/mL of MG and AuNPs@MG were evaluated. According to the standard practices, the results were analyzed and compared to the Assessment of Hemolytic Properties of Materials (ASTM F756-08 [50]), which classified the hemolytic activity into three classes: non-hemolytic (0–2% of hemolysis), slightly hemolytic (2–5% of hemolysis), and hemolytic (values higher than 5%) [28,51] For MG alone, it was found that for concentrations of 100, 10, and 1 µg/mL, hemolysis was found to be under 2%, while at the highest concentration (1000 µg/mL), it has a slightly hemolytic activity (hemolysis rate between 2 and 5% (Figure 7)), in agreement with previously reported results of MG [28]. On the other hand, AuNPs@MG had a hemolysis rate between 2 and 3% at 100, 10, and 1 µg/mL, making it a slightly hemolytic material at these concentrations. Previous studies have shown that AuNPs synthesized using green reducing agents such as Arabic gum present a low percentage of hemolysis compared to particles obtained using other non-green stabilizing agents [52]. One of these studies was carried out by Aldawsari et al., who used Arabic gum as a reducing and stabilizing agent to synthesize AuNPs. The hemolysis rate was 2% upon exposure to AuNPs with concentrations of 5–20 µg/mL [52].

#### 3.7.2. MTT Assay

**Figure 8:**
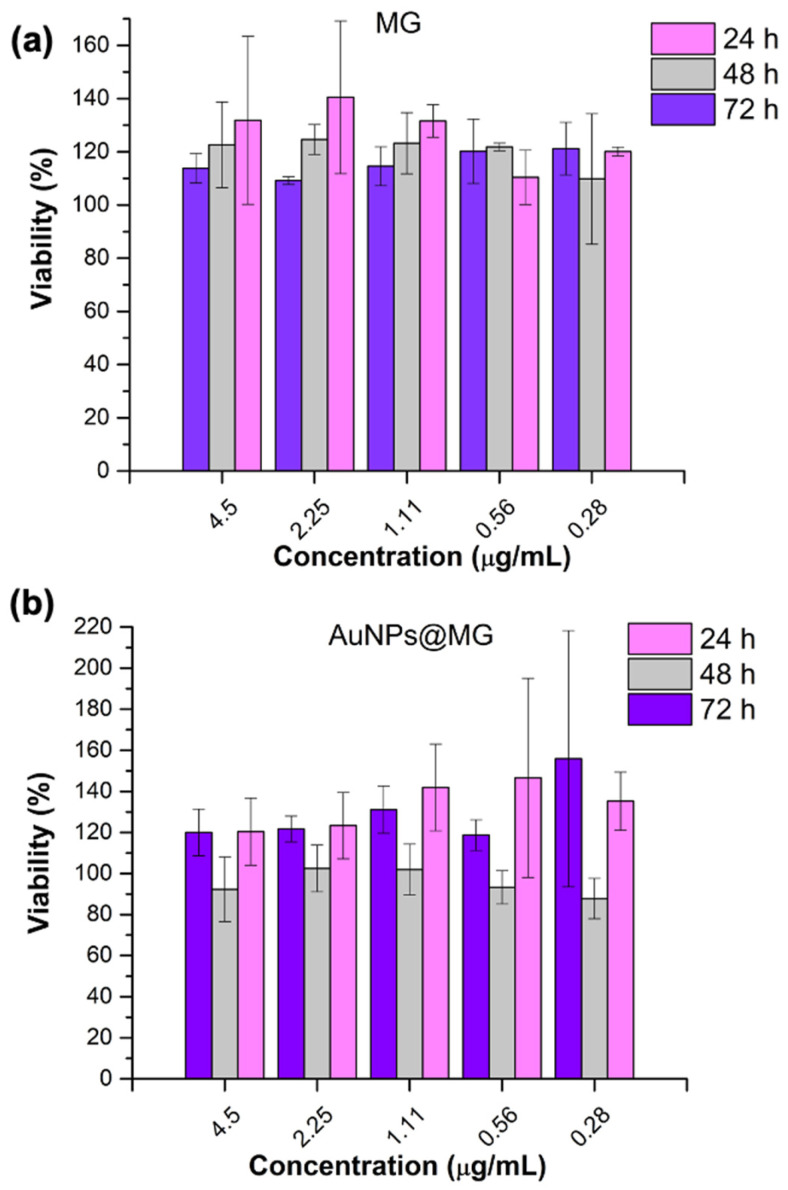
MTT assay of (**a**) MG and (**b**) AuNPs@MG (1:15).

The colorimetric technique with MTT (3-(4,5-Dimethylthiazol-2-yl)-2,5-Diphenyltetrazolium Bromide) evaluates the cell metabolic activity, specifically the activity of oxidoreductase enzymes located in the mitochondria. In non-affected cells (healthy cells) treated with the MTT reagent, through the enzymatic activity of oxidoreductase, the MTT is reduced to formazan crystals, which are water-insoluble and show a purple coloring [53]. The viable cell number can be associated with formazan in the assay. The cytotoxicity of AuNPs@MG and MG was evaluated with the treatment of 3T3L1 cells, observed using the MTT assay. The evaluation was conducted after 24, 48, and 72 h of incubation [28]. Figure 8 indicates that there is no statistical difference (*p* = 0.05) at all tested AuNPs@MG (1:15) and MG concentrations compared to the negative control (Figure 8). The results were compared to those obtained by Vijayakumar et al., who evaluated the cytotoxicity of gold nanoparticles stabilized with citrate, starch, and Arabic gum in MCF-7 cells. It was shown that AuNPs coated with Arabic gum did not cause a decrease in cell viability under the conditions evaluated. In summary, this assay showed that using mesquite gum as a reducing and stabilizing agent improved the biocompatibility of the AuNPs. Previous studies have shown that naturally derived gums improve the biocompatibility of NPs and copolymers [22,28].

#### 3.7.3. H&E Staining

**Figure 9:**
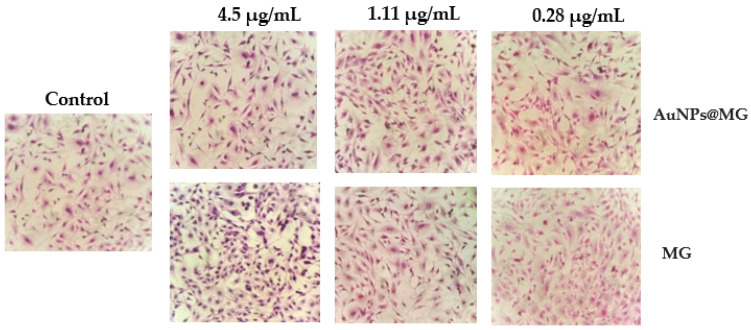
Optical microscope images of 40× of H&E staining at 24 h after 24 h exposure to AuNPs@MG (1:15), MG, and the control.

We evaluated the morphological differences in the 3T3L1 cells due to their treatments using H&E staining microscopy. Figure 9 displays optical images from an optical microscope at 40× after 24 h AuNPs@MG (1:15) and MG exposure. There were no clear cytoplasm or nucleus morphology changes at all the concentrations evaluated for AuNPs@MG and MG. These results correlate with the results obtained through MTT, in which there was no evidence of a decrease in cell viability at the same concentrations.

## 4. Conclusions

This study proposes a new green synthesis methodology for AuNPs using mesquite gum as a reducing and stabilizing agent. By offering an alternative to reducing and stabilizing agents that may be cytotoxic, this study contributes to a more sustainable and non-toxic approach to AuNP synthesis. The use of MG as a green agent is commendable, especially considering the supply issues faced by AG due to environmental challenges. Additionally, the AuNPs@MG are not cytotoxic, and they are stable at physiological pH, primarily attributed to the biocompatible nature of MG, which also provides stability to the AuNPs. The observation of low hemolytic activity for both MG and AuNPs@MG at specific concentrations is crucial in assessing their biocompatibility, particularly for intravenous administration. This finding enhances their potential applications in the biomedical field.

In conclusion, this study successfully highlights MG as a viable green and biocompatible reducing and stabilizing agent for AuNP synthesis. The biocompatibility, stability, and unique properties of AuNPs@MG make them promising candidates for various applications in nanomedicine and related industries. This research offers valuable insights into the potential of natural biopolymers for nanomaterial synthesis, promoting eco-friendly and sustainable approaches. The comprehensive conclusions and clear presentation of findings make this study a significant contribution to the field of nanotechnology and biomedicine.

## Figures and Tables

**Table 1:** Hydrodynamic diameter for AuNPs@MG at three weight ratios.

HAuCl_4_ Concentration (mM)	HAuCl_4_:MG Ratio	PdI	Size (d.nm) ± SE (%Volume)	
			1st Peak	2nd Peak
1	1:15	0.193	77.60 ± 42.1 (100%)	
1	1:26	0.292	15.53 ± 12.69 (100%)	
1	1:50	0.438	5.43 ± 1.69 (97.7%)	39.10 ± 17.86 (2.3%)

**Table 2:** Measurements of zeta potential for AuNPs@MG at three weight ratios.

HAuCl_4_ Concentration (mM)	HAuCl₄:GM Ratio	Zeta Potential (mV)
1	1:15	−30.9 ± 11.2
1	1:26	−26.4 ± 10.3
1	1:50	−26.2 ± 9.51

## Data Availability

The data presented in this study are available on request from the corresponding author.

## Supplementary Materials

The following are available online at https://www.mdpi.com/article/10.3390/polym15173533/s1, Figure S1. Size distribution of AuNPs@MG at 1:50 weight ratio (*W_MG_*/*W_Au_*) as obtained with DLS. Figure S2. STEM micrographs of AuNPs@MG obtained at a 1:15 weight ratio. Table S1. Comparison of the synthesis of AuNPs using AG documented in the literature and the method reported in the current work.
