# Supplementary material for: Green Synthesis of Mesquite-Gum-Stabilized Gold Nanoparticles for Biomedical Applications: Physicochemical Properties and Biocompatibility Assessment
Source: Polymers (Basel). 2023 Aug 24;15(17):3533. doi: 10.3390/polym15173533 (PMC10490394; doi:10.3390/polym15173533)
Supplement: Supplementary file 1 [file polymers-15-03533-s001.zip › polymers-2497072-supplementary.pdf]

polymers

MDPI

Supplementary Materials

Green Synthesis of Mesquite-Gum-Stabilized Gold Nanoparticles for Biomedical Applications: Physicochemical Properties and Biocompatibility Assessment

Ana M. Pinilla-Torres [1,†], Celia N. Sanchez-Dominguez [2,†], Karla Basilio-Bernabe [1], Paola Y. Carrion-Garcia [2], Jorge A. Roacho-Perez [2], Elsa N. Garza-Treviño [2], Hugo Gallardo-Blanco [3,*] and Margarita Sanchez-Dominguez [1,*]

[1] Grupo de Química Coloidal e Interfacial Aplicada a Nanomateriales y Formulaciones, Centro de Investigación en Materiales Avanzados, S.C. (CIMAV, S.C.), Unidad Monterrey, Apodaca 66628, Mexico; ana.pinilla@cimav.edu.mx (A.M.P.-T.); karlabasiliobernabe96@gmail.com (K.B.-B.)
[2] Departamento de Bioquímica y Medicina Molecular, Facultad de Medicina, Universidad Autónoma de Nuevo León, Monterrey 64460, Mexico; celia.sanchezdm@uanl.edu.mx (C.N.S.-D.); carriongarcia.paola@gmail.com (P.Y.C.-G.); alberto.roachoprz@uanl.edu.mx (J.A.R.-P.); elsa.garzatr@uanl.edu.mx (E.N.G.-T.)
[3] Departamento de Genética, Facultad de Medicina, Universidad Autónoma de Nuevo León, Monterrey 64460, Mexico
* Correspondence: hugo.gallardobl@uanl.edu.mx (H.G.-B.); margarita.sanchez@cimav.edu.mx (M.S.-D.)
† These authors contributed equally to this work.

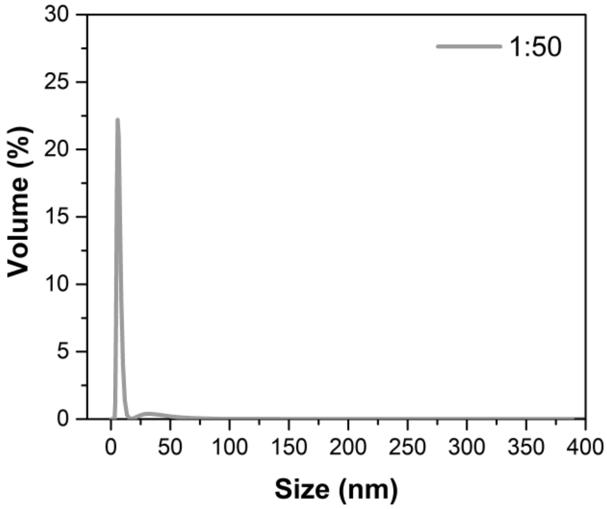

Figure S1. Size distribution of AuNPs@MG at 1:50 weight ratio (W_{MG}/W_{Au}).

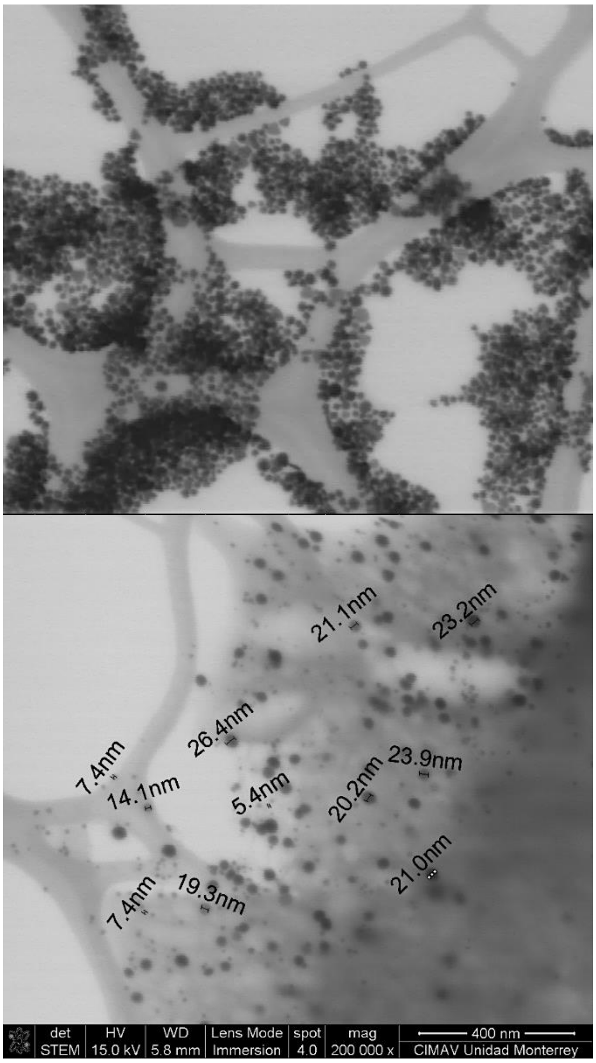

Figure S2. STEM Micrographs of AuNPs@MG obtained at a 1:15 weight ratio.

Table S1. Comparison of the synthesis of AuNPs using AG reported in the literature and the method reported here.

Authors	Reducing and stabilizing agent	Reaction time	Tempera-ture	Shape/ Average particle size	Observations	Refe-rence
**Pinilla et al.**	Mesquite gum	180 min	70°C	Globular NPs/18.3±7.3	We do not use toxic reducing and stabilizing agents<br>Stable at physiological conditions (PBS)<br>Biocompatible.<br>Hemocompatible	**This work**
**Ribeiro de Barros et al.**	$NaBH_4$ (reducing agent)<br>Arabic gum<br>(Stabilizing agent)	60 min	Room temperature	Spherical/ 5.4 nm	It is necessary to leave the mixture overnight at 4°C to ensure complete hydration of the gum.<br>Hemocompatibility was not evaluated.<br>$NaBH_4$ was used and it is cytotoxic.	[1]
**Gamal-El-deen et al.**	$P(CH_2NHCOOH)_3$ (Reducing agent)<br>Arabic gum<br>(Stabilizing agent)	10 min	80 °C	Spherical/ 21 nm	An additional chemical compound was used to reduce the gold.<br>Stability over time under physiological conditions was not evaluated<br>Hemocompatibility was not evaluated	[2]
**Wu et al.**	Arabic gum	240 min	55°C	Spherical/ 26.8nm	Stability over time under physiological conditions was not evaluated.<br>Hemocompatibility was not evaluated	[3]
**Iranpour et al.**	Ionic liquid D-glucosammonium	20 min	60 °C.	Spherical/ 25 nm	Ionic liquids are relatively high cost compared to other reducing agents.	[4]

	formate<br>(Reducing agent)<br>Arabic gum<br>(Stabilizing agent)				Hemocompatibility was not evaluated.	
**Gonçalves et al.**	$NaBH_4$<br>Ascorbic acid<br>(Reducing agents)<br>CTAB<br>Arabic gum<br>(Stabilizing agents)	360min	Room temperature	Rods	CTAB and $NaBH_4$ are cytotoxic<br>Long reaction time.<br>Hemocompatibility was not evaluated.	[5]

References

1. Ribeiro, de Barros H, Cardoso, MB, Camargo de Oliveira, C, et al (2016) Stability of gum arabic-gold nanoparticles in physiological simulated pHs and their selective effect on cell lines. RSC Adv 6:9411–9420. https://doi.org/10.1039/C5RA24858B
2. Gamal-Eldeen AM, Moustafa D, El-Daly SM, et al (2016) Photothermal therapy mediated by gum Arabic-conjugated gold nanoparticles suppresses liver preneoplastic lesions in mice. J Photochem Photobiol B 163:47–56. https://doi.org/10.1016/j.jphotobiol.2016.08.009
3. Wu C-C, Chen D-H (2010) Facile green synthesis of gold nanoparticles with gum arabic as a stabilizing agent and reducing agent. Gold Bull 43:234–240. https://doi.org/10.1007/BF03214993
4. Iranpour P, Ajamian M, Safavi A, et al (2018) Synthesis of highly stable and biocompatible gold nanoparticles for use as a new X-ray contrast agent. J Mater Sci Mater Med 29:48. https://doi.org/10.1007/s10856-018-6053-5
5. Gonçalves JP, da Cruz AF, Nunes ÁM, et al (2021) Biocompatible gum arabic-gold nanorod composite as an effective therapy for mistreated melanomas. Int J Biol Macromol 185:551–561. https://doi.org/10.1016/j.ijbiomac.2021.06.172
